# Imaging of Gastric Carcinomatosis

**DOI:** 10.3390/jcm10225294

**Published:** 2021-11-14

**Authors:** Raquel Saiz Martínez, Clarisse Dromain, Naik Vietti Violi

**Affiliations:** 1 MD Anderson Cancer Center Madrid, Department of Radiology, 28033 Madrid, Spain; 2 Department of Radiology, Lausanne University Hospital and University of Lausanne, 1015 Lausanne, Switzerland; Clarisse.Dromain@chuv.ch

**Keywords:** gastric cancer, peritoneal carcinomatosis, imaging, CT, MRI, PET/CT, PET/MR

## Abstract

Diagnosing the absence or presence of peritoneal carcinomatosis in patients with gastric cancer, including its extent and distribution, is an essential step in patients’ therapeutic management. Such diagnosis still remains a radiological challenge. In this article, we review the strengths and weaknesses of the different imaging techniques for the diagnosis of peritoneal carcinomatosis of gastric origin as well as the techniques’ imaging features. We also discuss the assessment of response to treatment and present recommendations for the follow-up of patients with complete surgical resection according to the presence of risk factors of recurrence, as well as discussing future directions for imaging improvement.

## 1. Introduction

With the emergence of possible treatment strategies for patients with peritoneal metastases (PM) of gastric origin, the role of imaging for the accurate staging of these patients is becoming increasingly important. In addition to evaluating the presence or the absence of PM, imaging has a key role when assessing its extent and distribution.

Although advances in imaging technology have allowed a significant increase in spatial and contrast resolution, depiction of peritoneal disease remains a challenge, on one hand due to the small size of the implants and their weak enhancement after contrast injection, while on the other hand due to the extensive surface area that may host tumour deposits.

Compared with PM from other types of cancer, such as colonic or ovarian cancers, gastric carcinomatosis has specific issues to overcome: (1) the translymphatic process of peritoneal metastases at early stage, with implants location into milky spots [1] in the greater omentum, mesenterium, and pelvic floor; (2) the infiltrative pattern with small size implants of the diffuse-type of gastric cancer (GC); and (3) the low FDG avidity of the mucinous and diffuse-type of gastric cancer.

This article reviews the imaging modalities used for gastric cancer detection and their performance depending to the histology sub-type and provides an overview of their imaging appearance. Assessment of response and follow-up recommendations after complete surgical resection, including the specific situation of patients with positive peritoneal washing cytology during the surgery without gross PM, are also discussed. 

## 2. Imaging Modalities

**Figure 1:**
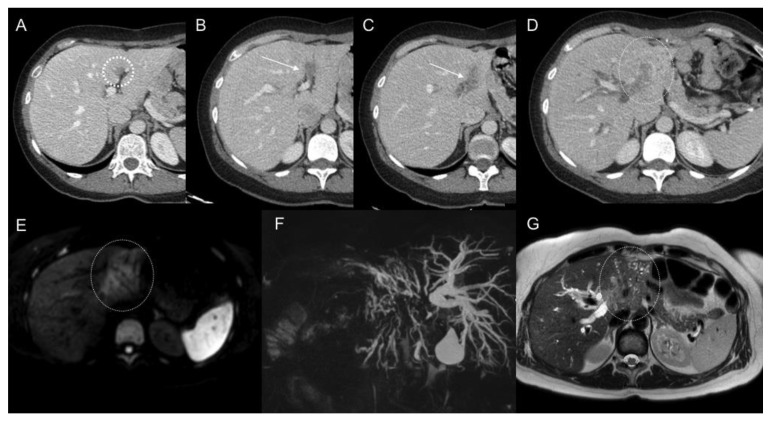
Focal soft tissue lesion in the deep part of the falciform ligament (circle), retrospectively identified in the first CT scan post-surgery (**A**) of a patient who underwent surgery because of gastric adenocarcinoma with positive peritoneal lavage. Progressive increase in size during the follow-up CT scans (**B**,**C**) involving the left portal vein and being mis-diagnosed as portal vein thrombosis (arrows). Further follow-up showed evidence a soft tissue infiltrating mass (oval) in left hepatic lobe (**D**) and DWI-b800 (**E**) and T2WI (**G**) MR images, with spread along periportal spaces and conditioning biliary obstruction as MRCP (**F**) shows.

Imaging of PM is challenging, as there are different imaging technical requirements that include: (1) the need for high spatial resolution due to frequently small lesion size; (2) high contrast resolution, as PM presents a low spontaneous contrast as well as a low contrast enhancement; and (3) the need to mitigate any bowel peristalsis artifact. Imaging modalities include computed tomography (CT), magnetic resonance imaging (MRI), and positron emission tomography (PET). None of them will fulfil all requirements, and radiologists will consider that during PM assessment. Imaging assessment of PM is known for a low inter-reader agreement and a limited level of confidence regarding the presence of a lesion. PM is unfortunately very often seen retrospectively after surgery or follow-up (Figure 1). The diagnostic performance of PM depends on radiologist experience, imaging modality, lesion morphology, location, and histology. Some practical recommendations need to be applied in order to improve PM detection and staging, including in cases of GC: (1) consider the peritoneal cavity as an individual organ; (2) use multi-plane reconstructions; and (3) have a specific review of the different peritoneal spaces, particularly meso and fat spaces.

### 2.1. Computed Tomography (CT)

**Figure 2:**
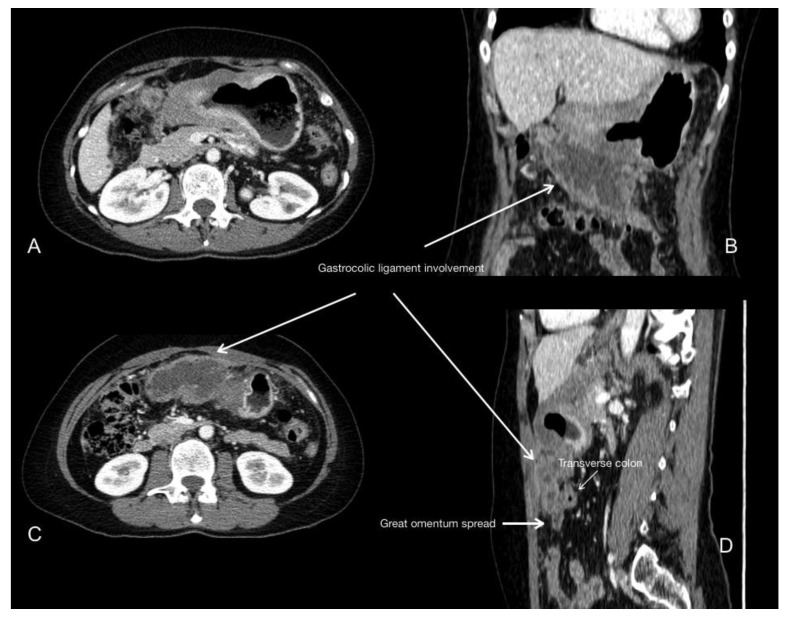
Primary gastric cancer involving distal body and antrum. Portal phase contrast-enhanced CT axial (**A**,**C**), coronal (**B**) and sagittal (**D**) images show the tumour spreading inferiorly along the gastrocolic ligament, reaching the transverse colon and involving the greater omentum.

CT remains the modality of first choice for peritoneal imaging, and it is included in ESMO guidelines for GC [2,3]. CT is fast, which limits motion artefacts, and it allows high spatial resolution (when performed in portal venous phase after intravenous contrast injection) with the possibility of performing coronal and sagittal reconstructions (Figure 2). However, it has limited soft tissue contrast, which limits the depiction of small PM lesions, particularly when adjacent to bowel structures or the mesentery, limiting its sensitivity for lesion detection and accuracy in staging PM, with underestimation compared with surgical Peritoneal Cancer Index (PCI) [4,5]. For that reason, laparoscopy remains the reference for PC staging. A large inter-reader variability has been reported, highlighting the importance of a reader’s expertise [6]. Additionally, agreement varies according to anatomical site. In patients with PC from ovarian origin, inter-reader agreement was good to excellent for the positive diagnostic of PM (k = 0.75–0.91), while it dropped significantly when considering small bowel locations (k = 0.12–0.29) [7]. The same results were confirmed in PC from gastric cancer [8]. CT sensitivity for PC is low to moderate, with wild variations, ranging from 23% to 76% [9,10]. Sensitivity is particularly low in cases of small lesion size (ranging from 11% in <5 mm lesion compared with 94% for >5 cm lesion) and in some difficult locations, including the mesentery, diaphragmatic cupola, and lesions located on the bowel wall [5,11]. In cases of GC, a review of four studies showed evidence of low sensitivity (14.3–59.1%), with high specificity (93.3–99.8%) for distant metastasis detection [12], with even lower sensitivity (28.3%) when considering PM only [13]. 

### 2.2. Magnetic Resonance Imaging (MRI)

**Figure 3:**
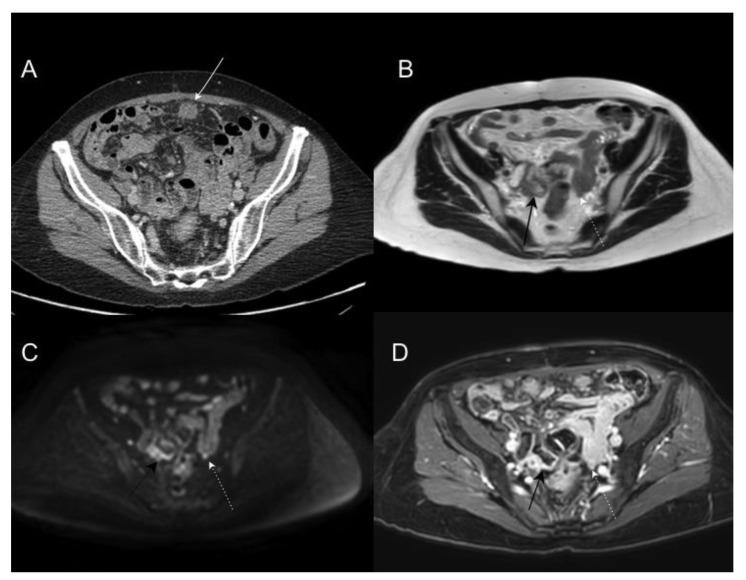
Portal phase contrast-enhanced CT axial image (**A**) shows mesenteric fat stranding and soft tissue mesenteric nodules (white arrow). In T2WI axial (**B**), DWI b1000 axial (**C**), and postGd DIXON axial (**D**) MR images, we can identify smaller soft tissue nodules, some of them laying on bowel surface (white dotted arrows) best depicted in DWI, and others with associated with focal bowel wall thickening (black arrows), with enhancement and restricted diffusion as a sign of serosal involvement.

MRI allows higher soft tissue contrast than CT, multiple post-contrast acquisitions (particularly late post-contrast phases), and the possibility of using diffusion weighted imaging (DWI). However, MRI is limited by longer examination time and the risk of motion artifacts (which can be reduced by the use of anti-peristaltic agents) [14]. Even more than in CT, the experience of the reader has shown to be important with the use of MRI, as evidenced by Kappa agreement for the presence of PM: k = 1.0 with experienced reader in both CT and MRI, while it was k = 0.58 for MRI and k = 0.73 for CT for the inexperienced reader [15]. MRI allows high sensitivity for PM detection, particularly in small size lesions [11,16] (Figure 3). Realization of late sequences after intravenous injection also improve lesion detection, with the possibility of selected tissue saturation as liquid (ascites, bowel liquid) and fat [7,11]. MRI seems to be superior to CT for the preoperative assessment of PCI, with a sensitivity and specificity of 90% and 95.5%, respectively [17]. Compared with CT, MRI also showed evidence of an ability to find PC in difficult locations, such as on the right diaphragm, mesentery, bowel, and pelvis [10,18]. Moreover, the use of T2-weighted imaging (T2WI) is of great interest when considering mucinous tumour subtypes, as PC typically appear with T2WI hyper-intensity, making this sequence very sensitive. It is of particular interest in the context of GC, as PM are frequently a small size lesion or infiltrative disease, with limited sensitivity to CT, as shown by De Vuysere et al. [8]. Their work showed evidence of a CT sensitivity for overall gastric tumour detection that was 83.3% vs. 100% for MRI, with a significant difference when considering tumour infiltration of the mesentery, small bowel, and PM (0% vs. 100% for CT vs. MRI, respectively). Realization of whole-body MRI has been proposed to allow for the assessment of primary tumour and lymph node involvement and distant metastasis for GC [8]. Nonetheless, MRI is not currently recommended as a first-line imaging modality for PM from GC but rather for the staging before surgery or as a problem-solving tool. 

### 2.3. Positron Emission Tomography (PET)-CT

**Figure 4:**
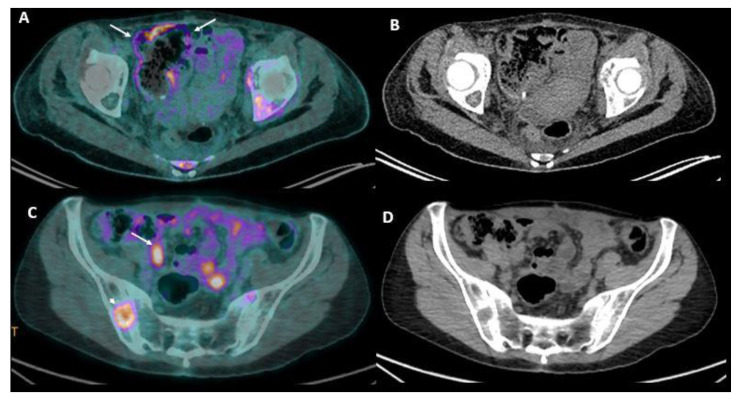
A 45-year-old female patient with peritoneal carcinomatosis from diffuse cell type gastric cancer. PET-CT fusion (**A**,**C**) with corresponding native CT images (**B**,**D**) shows evidence of linear FDG uptake of the caecum surface (**A**, arrows) and focal uptake within the pelvic peritoneum (**C**, arrow), both corresponding to peritoneal carcinomatosis. Note the presence of focal FDG uptake in iliac bone corresponding to a biopsy-proven metastasis (**C**, arrowhead). PET: position emission tomography.

**Figure 5:**
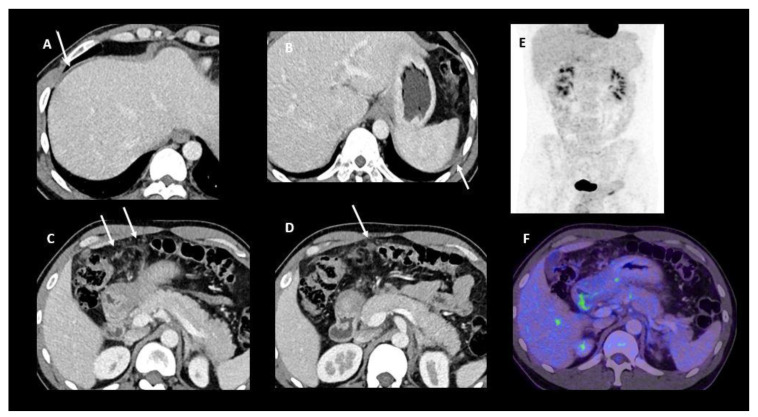
Contrast-enhanced FDG PET/CT performed for initial staging of a newly diagnosed diffuse-type gastric cancer with signet ring cells. Contrast-enhanced CT images (**A**–**D**) show a small amount of ascites around the liver and the spleen and a micronodular infiltration of the great omentum. No FDG tracer uptake was depicted on either MIP projection (**E**) or fused PET/CT image (**F**). A laparoscopic exploration confirmed the presence of a peritoneal implant located on the right and left diaphragm and around the round ligament.

The use of 18F-fluorodesoxyglucose (18FDG) PET combined with non-contrast CT (PET-CT) is helpful for characterization and tumour staging in cases of PM. It allows a very high lesion contrast and the identification of lesion location that is frequently missed on CT (Figure 4). It is particularly useful for the assessment of extra-peritoneal disease. PET-CT is currently indicated in cases of tumour biomarker elevation without evidence of tumour. Inter-reader agreement is known to be good to excellent for PM lesion assessment, including in the context of GC [19]. As for CT, the sensitivity of PET evidence varies, ranging from 57% to 86.4% [14,20]. However, detection sensitivity decreases in lesions < 5 mm [21]. Additionally, PET-CT sensitivity depends on histological subtype, which is related to variation in FDG uptake. In diffuse mucinous and signet ring cells, GC subtype tumours showed evidence of lower FDG uptake, and thus lower sensitivity in lesion detection, compared with non-mucinous intestinal type (Figure 5). This is explained by the low cellular density and quite inert mucus seen in these tumour subtypes [22]. 

PET-CT seems to be superior to CT for mesentery and bowel locations [15]. PM will manifest as focal FDG uptake. However, a thin and linear fixation, following peritoneum folds or plain organ capsule, is also suggestive of PM, suggesting an infiltrative peritoneal involvement [15] (Figure 4). False negatives in cases of GC are related to a low or no FDG uptake by the tumour (depending on histological subtypes) as well as being related to micronodular infiltration, improving patient selection for 18F-FDG PET scanning in the staging of GC (Figure 5). False positive cases are frequently related to physiological tracer uptake in the bowel and urinary track [16]. Due to the mentioned limitations, sensitivity for PM detection in GC seems to be lower than that of CT and MRI [19,23]. PET-CT has been also suggested for the evaluation of recurrence after complete surgical resection (CSR) [24].

### 2.4. Imaging Combination

As none of the described imaging tools are ideal for PM detection and staging, some authors suggested a combination of them, taking advantage of the strengths of each one. In that sense, PET-CT coupled with intravenous contrast-enhanced CT has been investigated with contradictory results [25,26] (Figure 5). Lin et al. recommended the combination of thoraco-abdomino-pelvic CT with abdomino-pelvic MRI for pre-operative assessment of PM (including from GC origin) due to higher inter-reader agreement, true positive rate, and smaller error in PCI evaluation when compared with CT alone [6]. 

## 3. Imaging Features of Gastric Carcinomatosis

Imaging assessment of peritoneal disease requires a good knowledge of the pathobiology of gastric carcinoma and of the mechanisms of peritoneal spread [27]. According to Lauren classification [28], there are two major histological types of gastric adenocarcinoma: intestinal and diffuse. The latter includes the signet ring cell subtype. In both types, the mucinous component may be present. The intestinal type is well or moderately differentiated and is associated with a better prognosis. On the other hand, the diffuse type is poorly differentiated and is typically a high-grade tumour, associated with a worse prognosis. The main clinical difference lies in their recurrence patterns, with the diffuse type being more prone to peritoneal dissemination, especially when the serosa is involved, while the risk of liver metastases is higher in the intestinal type. As for the mechanisms of spread, peritoneal tumour seeding commonly occurs transperitoneally within the peritoneal cavity, but GCs are also able to spread within the subperitoneal space between the layers of the peritoneum and along the perigastric ligaments, mesentery, or omentum. 

**Figure 6:**
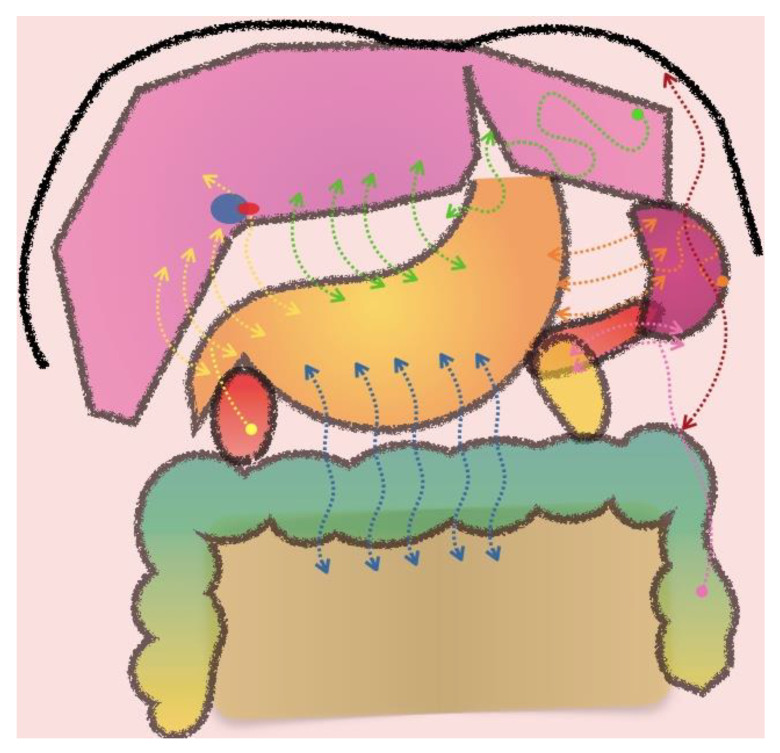
Patterns of peritoneal spread depending on the location of the primary tumour. Hepatoduodenal ligament (yellow arrows). Gastrohepatic ligament (green arrows). Gastrosplenic ligament (orange arrows). Splenorrenal ligament (pink arrows). Gastrocolic ligament (blue arrows). Frenocolic ligament (red arrows).

**Figure 7:**
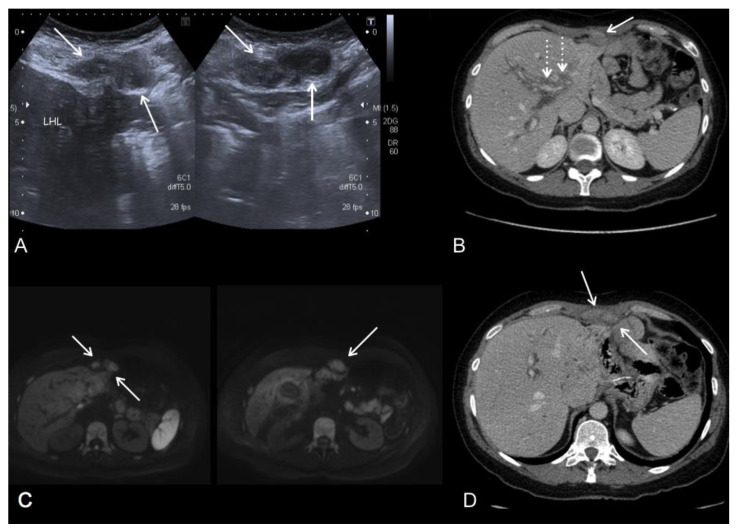
Imaging follow-up of a 50-year-old woman who underwent total gastrectomy for diffuse gastric adenocarcinoma. US (**A**), portal phase contrast-enhanced CT axial (**B**,**D**) and MR-DWI b800 (**C**) images show an epigastric implant (arrows) invading the abdominal wall. Disease extended to the left intersegmental fissure and continued along the falciform ligament, reaching the left hepatic lobe and the abdominal wall. Note the right periportal tumour dissemination (dotted arrows).

**Figure 8:**
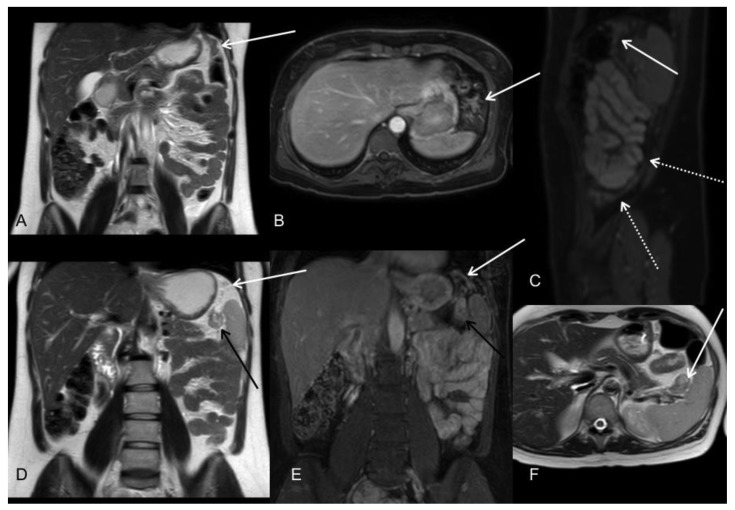
Patient with diagnosis of signet cell gastric adenocarcinoma located in the greater curvature of the upper body. MRI at initial staging is shown. T2WI coronal (**A**,**D**), postGd DIXON axial (**B**), sagittal (**C**), and coronal (**E**), T2WI axial (**F**). MR images demonstrated tumoral implants in the gastrosplenic (black arrows) and phrenocolic ligaments (white arrows). Subphrenic involvement is better seen in coronal and sagittal planes. Note also the involvement of the posterior parietal peritoneum in sagittal plane (white dotted arrows).

The primary site of GC has crucial surgical implications. Moreover, it is a blind spot for surgeons during staging laparoscopy, increasing the importance of the role of imaging in accurate assessment. The anatomic location of the primary tumour in the stomach can help to predict the pattern of disease spread (Figure 6) [29]. Tumours located along the cardia, lesser curvature, and antrum spread more often along the gastrohepatic ligament, while tumours located in the pylorus and antrum usually spread along the hepatoduodeal ligament. The locoregional ligaments’ tumour involvement can result in the disease extending into the liver through the venous ligament and, eventually, reaching the periportal space by following the branches of the portal vein, which can be seen as enhanced soft tissue and/or restricted diffusion along them in MR images (Figure 1). The tumour may eventually extend to the left intersegmental fissure and continue along the falciform ligament to reach the anterior abdominal wall (Figure 7). Tumours in the fundus and along the greater curvature of the upper body of the stomach spread along the gastrosplenic ligament and less frequently along the splenorenal ligament. Tumours spread from there to the perisplenic and left subphrenic spaces, where they are depicted as nodules, masses, or thickening and enhancement ± restricted diffusion on MR images involving the left hemidiaphragm. Coronal and sagittal planes are very useful for evaluating and confirming the subphrenic location of metastases to this region (Figure 8). Tumours located along the greater curvature may spread inferiorly along the gastrocolic ligament to reach the transverse colon and the transverse mesocolon and may then involve the greater omentum (Figure 2). Involvement of the greater omentum fat is able to occur both via a subperitoneal and transperitoneal route, and it can manifest as anything from a subtle fat stranding and/or nodularity to a dense omental cake, depending on the degree of involvement.

The presence of ascites is one of the most common manifestations of gastric carcinomatosis [30]. A recent study found that the amount of ascites was an independent risk factor for PM in GC patients who did not show distant metastasis or PM on CT [31]. The authors reported that an ascites volume of >50 mL in GC patients in the absence of liver cirrhosis or chronic renal insufficiency is highly suggestive of PM. Transperitoneal spread via ascites facilitates tumour cells’ deposition and multiplication in areas of stasis or in areas where a large amount of peritoneal fluid is absorbed. Thus, the key spots for early tumour deposition include the pouch of Douglas, sigmoid colon and its mesentery, terminal ileum, right paracolic gutter, posterior right subhepatic space, right subphrenic space, and the omentum. Each of them should be evaluated with great care and attention, looking out for peritoneal metastases. 

**Figure 9:**
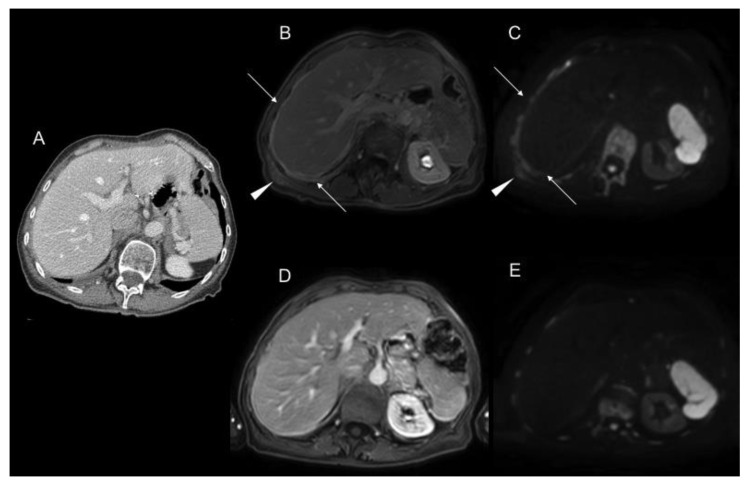
A 69-year-old female patient with diffuse gastric adenocarcinoma treated with total gastrectomy who presented progressive elevation of CA19.9 tumour marker during the follow-up. Portal phase contrast-enhanced CT axial image (**A**) show no findings. In initial MR images postGd DIXON axial (**B**) and DWI b1000 axial (**C**) we can see abnormal perihepatic enhancement and restricted diffusion (arrows), with involvement of the right dorsal muscle (arrowheads), consistent with tumour recurrence. Only a subtle perihepatic enhancement can be seen in the first MR images (**D**,**E**) post neoadjuvant chemotherapy.

**Figure 10:**
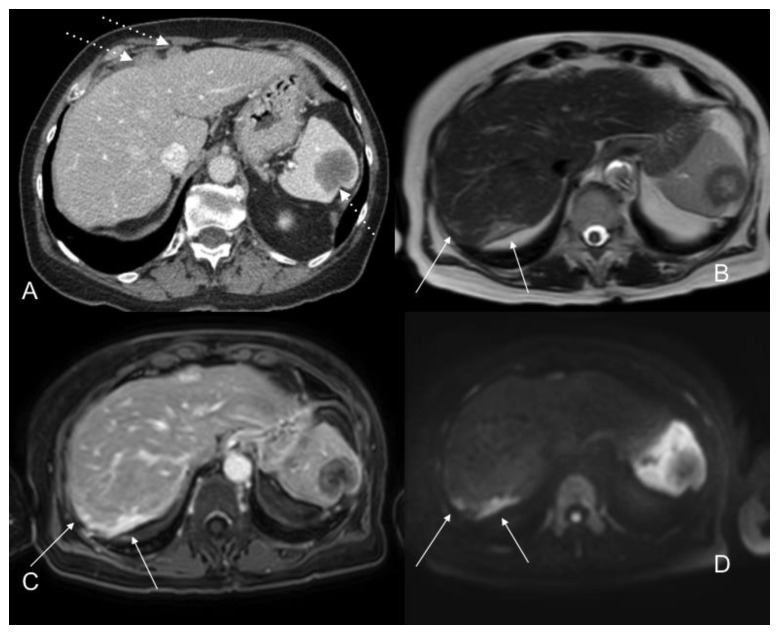
Portal phase contrast-enhanced CT axial images (**A**) show nodular anterior perihepatic implants as well as a splenic implant (white dotted arrows). T2WI axial (**B**), postGD DIXON axial (**C**), and DWI b800 axial (**D**) MR images also show extensive subcapsular involvement on the posterior surface of the right hepatic lobe (white arrows), not visible on CT scan. This location is a blind spot in exploratory laparoscopy.

Right subphrenic and subhepatic spaces involvement may present themselves as large nodules or masses, which are often well seen in CT scan and MR images. However, they can also simply present as enhancement, with or without associated thickening, which is usually less obvious or non-detectable in a CT scan and is better seen on MR images (Figure 9). On MRI, they are often correlated with high signal intensity on the fat suppressed T2 weighted images and with restricted diffusion (Figure 10). Detecting lesions in these locations is very important for clinical and surgical management, especially in the subhepatic space, as this location is a blind spot in exploratory laparoscopy. 

The parietal paracolic peritoneum can appear irregular and thickened with or without associated implants, or it can just show contrast enhancing with or without associated restricted diffusion on MRI. Frequently, the adjacent ascending or descending colon is involved. 

**Figure 11:**
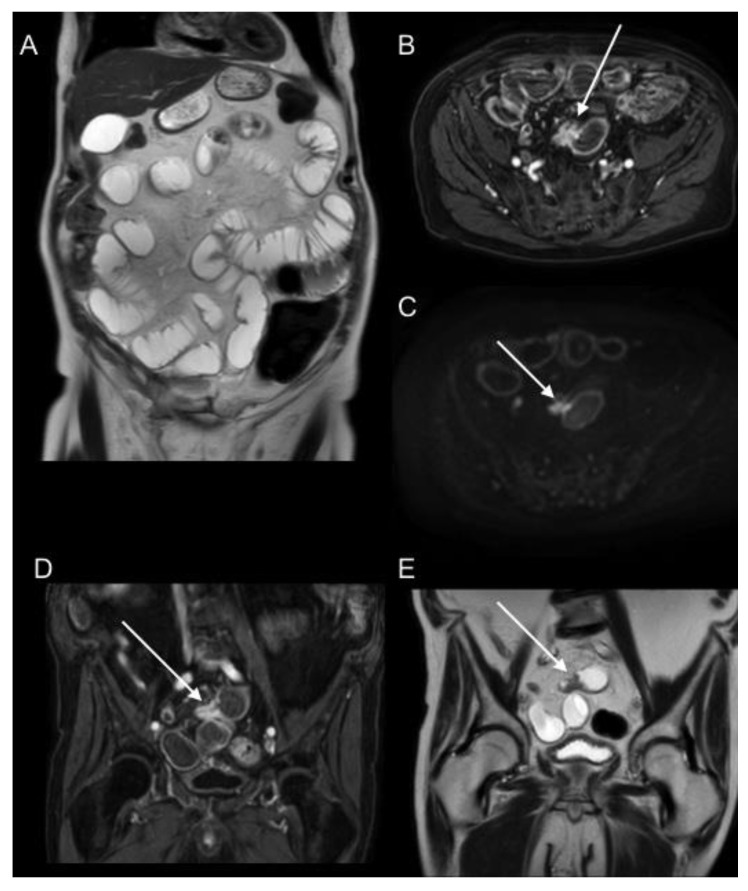
T2WI coronal and axial (**A**,**E**), postGD DIXON axial and coronal (**B**,**D**), and DWI b1000 axial (**C**) MR images show an intestinal obstruction due to ileal serosal implant (arrows).

Evaluation of small bowel mesentery and serosa involvement is not an easy task. Special attention should be paid to the ileocecal area, the recto-sigmoid colon, and the ligament of Treitz, as they are preferential areas of involvement in peritoneal carcinomatosis [32]. Early mesenteric tumour involvement may only be depicted by ill-defined enhancement and/or subtle changes in mesenteric fat density/intensity, usually better seen in CT images. In more advanced cases, we can observe increased thickness and soft tissue mesenteric masses causing separation and angulation of the small bowel and/or focal or diffuse bowel wall thickening as a sign of serosa involvement, which all can lead to retraction of the small bowel, drawing the bowel loops together against the posterior abdomen. Serosal peritoneal metastases are difficult to assess; they are especially unnoticed if limited to a few small tumour nodules on the bowel surface (Figure 3). They can also manifest as wall focal enhancement with/without associated restricted diffusion on MR-DWI images, or as increased thickness, associated nodules or masses, or changes in bowel diameter. Eventually, a serosal tumour produces partial or complete obstruction of the small bowel or colon (Figure 11).

**Figure 12:**
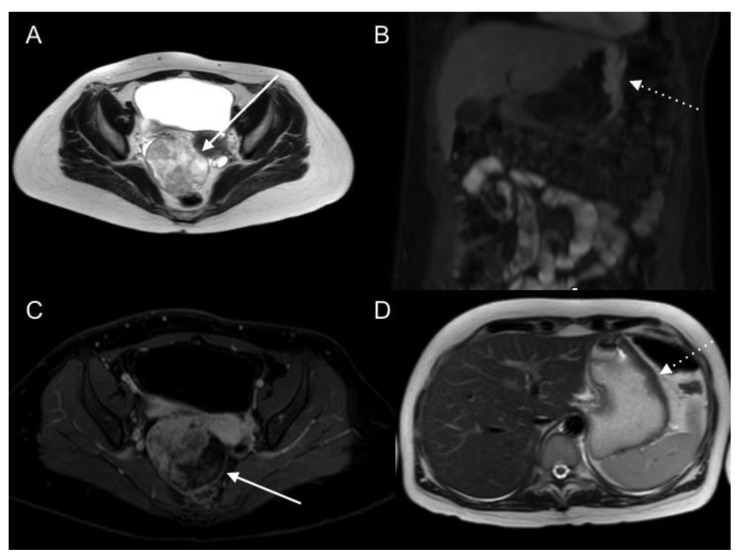
Patient in study for right adnexal mass. T2WI axial (**A**) and postGd DIXON axial (**C**) MR images of the pelvis show a markedly heterogeneous right adnexal mass with necrotic/cystic changes (arrows). DWI b1000 coronal (**B**) and T2WI axial (**D**) MR images of the upper abdomen show an abnormal thickening with restricted diffusion of the greater curvature of the gastric body (dotted arrows), suggesting the probable metastatic nature of the adnexal mass. Primary signet cell type gastric adenocarcinoma with ovarian metastases was confirmed.

The pelvis is also a commonly involved area. One can observe fluid, thickening, and enhancement ± restricted diffusion on MR images of the sidewall peritoneum with or without associated tumour nodules in variable size, with or without compression or infiltration of the urinary bladder, the rectum, or the uterus. Ovarian metastases from gastric adenocarcinoma (called as Krukenberg tumours) are usually of the signet ring cell histologic type and may be due to intraperitoneal spread of locally advanced GC or lymphatic spread in the absence of serosal gastric invasion. They are frequently the first manifestation of peritoneal dissemination in female patients (Figure 12). Ovaries can be uni- or bilaterally enlarged to a variable size, including massively enlarged ovaries that extend into the lower or mid abdomen, often with a heterogeneous density/intensity, including possible large cystic/necrotic changes. 

**Figure 13:**
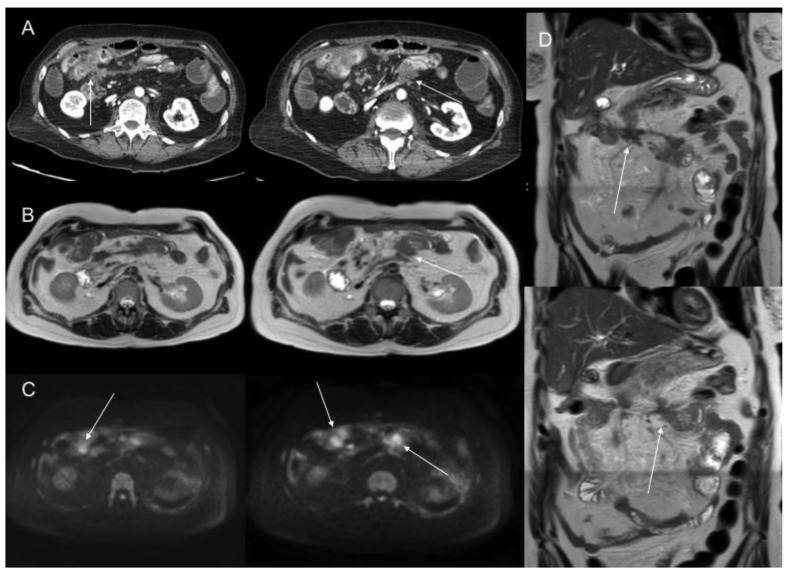
**A** 61-year-old male patient with peritoneal carcinomatosis from diffuse-type gastric cancer. Contrast-enhanced CT axial images (**A**) and T2WI axial and coronal (**B**,**D**) and DWI b1000 axial (**C**) MR images show mesenteric fat stranding and soft tissue nodules with multifocal infiltration and retraction of small bowel segments.

**Figure 14:**
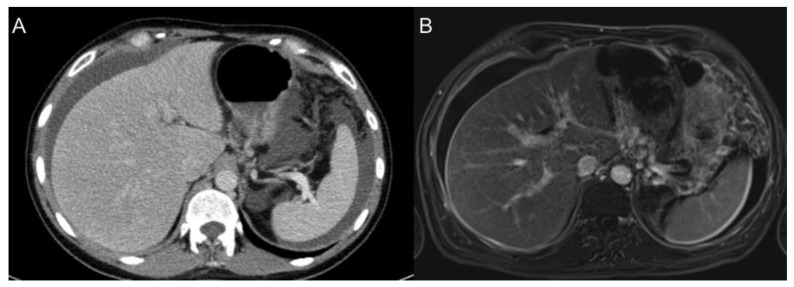
Gadolinium-enhanced MR sequences (**B**) are very efficient for showing supramesocolic peritoneum involvement. Contrast-enhanced CT (**A**) does not show the diffuse parietal and visceral peritoneal involvement.

Imaging is also key in identifying lesions that can limit or contraindicate potential surgery, such as: (1) lesions associated with an increased risk of incomplete tumour resection by identification of concerning radiologic features (Table 1); attention should be paid to the three more frequent sites for unresectable lesions—the small bowel and its mesentery (Figure 13), the porta hepatic, and the pelvis [33]; (2) lesions that require surgical subspecialty expertise because of their anatomic site—for example, diffuse spread of the liver capsule or of the diaphragm (Figure 14), multifocal lesions of the mesentery, involvement of the pelvic sidewall and vascular spaces, and abdominal wall involvement; and (3) occult peritoneal metastases—for example, lesions not visible during laparoscopy located in the Morrison pouch or posterior surface of the liver (Figure 10).

Imaging can be of great help in patient preoperative selection, avoiding surgery in patients whose tumours are too extensive for adequate surgical cytoreduction and identifying potential complications. Systematic lecture, including radiological PCI, great attention to details, and an experienced radiologist are key elements for achieving a proper interpretation of the examinations. Although it is a difficult task with frequent underestimation due to small and/or infiltrative lesions [34,35], radiologists should make the effort to calculate radiologic PCI during their preoperative workups. The PCI overestimation is infrequent and mainly due to benign infiltration of the fat. This means that a high CT/MRI-PCI is reliable and should be taken into consideration for the selection of patients for surgery. Conversely, a low CT/MRI-PCI has a more limited diagnostic accuracy. In these cases, and depending on other clinical factors, the benefit of an additional laparoscopy should be discussed. 

## 4. Assessment of Response

Although the optimal treatment of GC is not established, neoadjuvant chemotherapy is frequently used, including in cases of PM, before CRS and HIPEC [36,37]. In cases of (neo)-adjuvant chemotherapy, accurate differentiation between responders and non-responders is mandatory for further treatment decisions [38]. Assessment of the response is usually multimodal, combining imaging, endoscopy (primary tumour), and laparoscopy (for PM) [39]. Usually, an assessment of dimensional tumour changes using the Response Evaluation Criteria in Solid Tumors (RECIST) guidelines is used [40]. However, these criteria appear to be limited in GC evaluation. Indeed, the primary tumour is frequently not measurable based on infiltrative involvement of the gastric wall, and PM from GC is frequently a microscopic diffuse infiltration of the peritoneum rather than a focal lesion. As RECIST requires a measurable lesion (e.g., ≥1 cm lesion) for response assessment, it is not applicable under those conditions. Moreover, lesion dimension in the peritoneum is frequently affected by lesion shape irregularity, variation in visceral distention, limitation in the differentiation between residual tumour, and treatment-induced fibrosis [41]. Assessment of the presence and amount of ascites seems to be particularly important in GC, as it is correlated with worse prognosis [42]. However, it is not a measurable variable. Proposed alternatives to RECIST in GC include endoscopic evaluation of the primary tumour and laparoscopic evaluation of PM. Quantitative biomarkers, including changes in perfusion parameters on CT or MRI and changes in primary or FDG uptake, have been proposed [43,44]. DWI is also of interest, primarily because tumour and metastasis ADC values have been shown to be significantly higher in responders than in non-responders [41,45]. The ADC increase is explained by the presence of necrosis or fibrosis in cases of successful treatment [46]. However, these quantitative imaging biomarkers are not currently validated for routine clinical application. Thus, there is an unmet need for an accurate imaging tool for the assessment of response after (neo)adjuvant treatment response in patients with PM from gastric cancer. 

## 5. Follow-Up Imaging Recommendation after Surgical Resection

The development of peritoneal recurrence in patients with gastric cancer is associated with a poor prognosis and a reduced quality of life due to abdominal pain and/or distension and potential complications including bowel obstruction or refractory ascites. More than 50% of gastric cancer patients develop peritoneal recurrence following curative resection [47]. The identification of risk factors for peritoneal recurrence after complete surgical resection is very important in order to select those patients who can potentially benefit either from more aggressive therapeutic approaches, such as the combination of complete cytoreduction surgery with Hyperthermic Intraperitoneal Chemotherapy (HIPEC) or from less aggressive management, such as the use of Pressurized Intraperitoneal Aerosol Chemotherapy (PIPAC) [48,49]. The risk of peritoneal recurrence of gastric cancer is particularly high in patients with diffuse–mixed tumours, primary infiltration of the serosa, R1 surgical resection, and the presence of free cancer cells in the peritoneal lavage fluid [50,51]. Survival of patients with positive peritoneal cytology, in the absence of macroscopic peritoneal dissemination, and patients with macroscopic peritoneal dissemination is nearly the same [49].

Early detection of recurrence is the main goal of follow-up, in order to administer treatment to stop disease spread and to improve quality of life and survival when possible. There is still controversy surrounding the effectiveness of intensive follow-up of gastric adenocarcinoma patients following potentially curative resection, though there is no evidence that it improves survival outcomes [52,53,54]. However, many patients are uncomfortable with minimal and no follow-up, and continuous improvement of oncologic treatment increases the need for an early effective detection of peritoneal recurrences. The methods of follow-up include clinical examination, biochemical analyses, and radiological tools. There is not universal consensus on the radiological follow-up of gastric cancer patients. Among all of these methods, contrast-enhanced CT and FDG-PET-CT have been shown to have the best accuracy for exhibiting recurrence [53]. FDG-PET-CT is particularly useful when the recurrence is suspected clinically because of its high positive predictive value [54,55]. However, similar to initial staging, FDG-PET-CT is not recommended in the follow-up of diffuse-type mucinous adenocarcinoma and carcinomas containing signet ring cells that display low detectability on PET-CT. Some studies have reported the superiority of MR in preoperatively detecting peritoneal disease; nevertheless, no data exist regarding follow-up imaging. Whatever the imaging method used, follow-up image interpretation requires a comparison with post-operative images, with the goal of differentiating post-surgical changes from new lesions.

**Figure 15:**
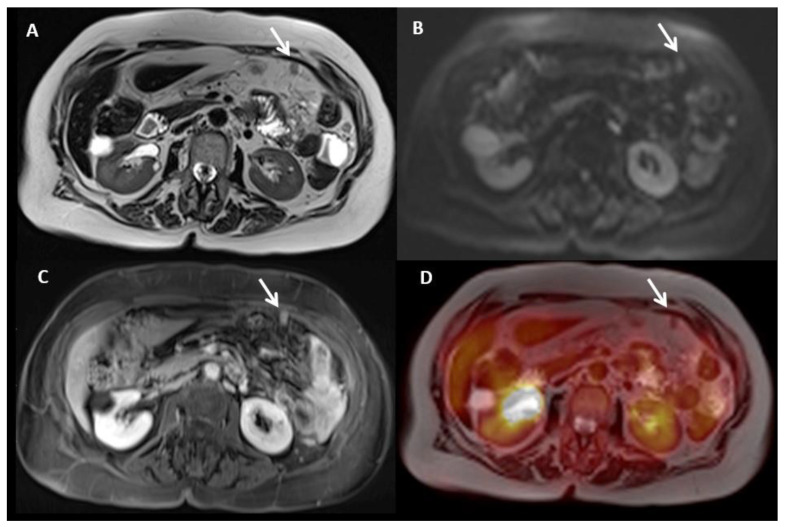
A 69-year-old female patient with peritoneal carcinomatosis from diffuse-type gastric cancer, including a 2 cm lesion located in the greater omentum. Lesion was visible (arrows) on T2WI (**A**), DWI (**B**), and contrast-enhanced T1WI (**C**) and was negative on PET (**D**).

The timing of follow up is also under debate. Imaging follow-up should be carried out in patients who are candidates for further treatment, and imaging should be tailored to the individual patient, taking into account the possibility of treatment and the risk of recurrence, with more frequent imaging follow-up in the initial T4 stage, histopathological diffuse-type, R1 resection, and positive peritoneal cytology (Figure 15). 

## 6. Future Directions

### 6.1. PET/MR

Both MRI and PET/CT have been shown to be useful in the preoperative staging of peritoneal metastases. In particular, diffusion MRI has been shown to provide better diagnostic performance than CT or PET/CT, with good correlation with surgical PCI [56,57]. However, both imaging modalities have limitations. PET/CT is limited by the variable physiological FDG uptake, which depends on the subtype of GC, and PET/CT is of limited value for small size implants. MRI is limited by the presence of artefacts—in particular, inhomogeneous fat suppression and physiological motion, such as bowel movements, which impair image quality and diagnostic accuracy.

**Figure 16:**
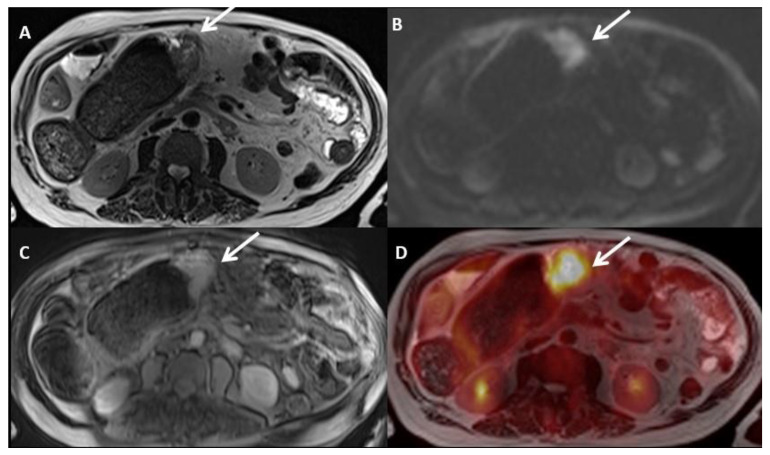
A 73-year-old male patient with peritoneal carcinomatosis secondary to gastric tubular adenocarcinoma, including a 4 cm lesion in the surface of the transverse colon. Lesion is visible in all images (arrows), including T2WI (**A**), DWI (**B**), contrast-enhanced T1WI (**C**), and PET (**D**).

PET/MRI is a recent integrated imaging modality that combines the anatomic, high soft-tissue contrast and functional capabilities of MR imaging together with molecular PET information. Depending on the histological type of the tumour, both techniques are often complementary in the detection and characterization of peritoneal carcinomatosis [58], with similar or better diagnostic performance than PET/CT, as evidenced by the more than 16 different cancer types [59,60] (Figure 15 and Figure 16). Only a few studies have evaluated the performance of PET/MRI in GC and/or peritoneal carcinomatosis [61,62,63]. Zheng et al. prospectively evaluated 30 patients newly diagnosed with localized GC, with both FDG PET/CT and FDG PET/MRI performed within the same day. They found similar accuracy but a significant higher lesion conspicuity for PET/MR T2WI images than for PET-CT images. 

A recent paper evaluated the value of FDG PET/MRI for assessing the extent of peritoneal metastases in 34 patients with ovarian and endometrial cancer [64]. They found a close correlation between PCI evaluation using PET/MRI, DW-MRI alone, and surgery. However, PET/MRI was superior to MRI alone in the evaluation of high tumour burden and tumour implants located close to the small bowel serosa. The exact role of PET/MRI in gastric carcinomatosis remains undetermined, but these promising results justify similar study in a larger cohort of GC patients with peritoneal carcinomatosis.

### 6.2. Novel PET Radiotracers

FDG PET imaging is limited in particular to early stage, diffuse-type, and mucinous tumours that are not FDG avid. 18F-fluothymine (F-18)FLT is an alternative radiotracer to FDG and reflects the activity of thymidine kinase 1 and has been developed to image cellular proliferation. A previous report identified that (F-18)FLT-PET/CT, despite low SUV, has a higher sensitivity than FDG-PET in GC, regardless of the histological type [65,66]. Honma et al. [67], in a proof-of-concept study, explored the sensitivity of 18F-fluothymine (F-18)FLT PET/CT for detecting PM from GC previously identified by other imaging modalities. A total of 19 patients, including 15 patients with diffuse-type, were analysed. PM was detected by (F-18)FLT-PET/CT in 14 of 19 patients, with a sensitivity of detection evaluated at 73.7% for PM, 100% for the primary tumour, and 42.7% for lymph nodes. One promising indication for FLT PET/CT is diffuse-type GC with a high number of signet ring cells, which has shown 100% sensitivity compared with 69% with FDG-PET [65]. Further studies including patients with CT and/or MRI occult PM implants will be of interest for better investigating the clinical utility of this new tracer for PM detection and staging.

FAPIs (fibroblast activation protein inhibitors) are a new class of radiotracers with highly promising results for detecting tumours that are characterized by a strong desmoplastic reaction and overexpressed FAP in their stroma. Qin et al. [68] prospectively compared 68Ga-DOTA-FAPI PET/MR and 18F-FDG PET/CT in 20 patients with GC. FAPI-PET provided excellent contrast, with higher tracer uptake, and was superior to FDG-PET in visualizing the primary tumour (100% vs. 71.43%) and PM (100% vs. 40%). FAPI-PET was also superior in visualizing abdominal lymph node metastases. Similar results have been shown for DOTA-FAPI PET/CT by Zhao L et al. in a cohort of 46 patients, including 16 patients with diffuse type of peritoneal carcinomatosis. They showed a significant difference in tracer uptake of peritoneal implants from GC, with a median SUV of 3.4 for FDG-PET to 8.5 for DOTA-FAPI-PET/CT [69]. FAPI PET/CT also shows promise for monitoring response to treatment in patients with peritoneal carcinomatosis from GC [70].

### 6.3. Radiomics and Deep Learning

The radiomics concept is based on the fact that images are more than pictures and that each image contains a lot information that cannot be captured by the human eye. Indeed, images are pixels containing data with which it is possible to conduct quantitative statistical analysis, such as texture analysis—a mathematical model that represents the spatial variations of an image. An alternative method is the deep learning (DL) approach, which is a type of artificial intelligence that automatically learns critical feature representations from sample images (training set) in order to put forward a prediction for new and unseen samples. Several studies have evaluated radiomics and deep learning for predicting occult peritoneal metastases in GC.

Liu et al. [71] retrospectively extracted radiomics features from preoperative portal phase CT scans in 233 patients (158 for training and 75 for validation). Among the 539 radiomics features extracted from 2D regions of interest of the gastric tumour, 52 were selected based on their higher reproducibility and redundancy. Six radiomics features differed statistically between GC with and without PM. However, the radiomics model remains inferior to the clinical–pathological model, including tumour location, pathology type, and degree of differentiation. Kim et al. [72] found that GC patients with PM showed significantly higher entropy, a biomarker of the irregularity of the structure, when considering textural features. When 7.14 was used as the cut off value of entropy in the validation study, specificity and sensitivity for the diagnosis of PM were 90% and 80%, respectively. Jiang et al. [73] evaluated deep learning in a large training cohort of 1225 patients with GC who underwent surgery, with an independent validation of two independent cohorts of 753 patients in total. In the training cohort, 135 patients had PM from GC (11%). The model achieved a sensitivity of 83.7% and a specificity of 92.8% for predicting the presence of PM. This performance was confirmed in the two validation cohorts, with sensitivities of 75.4% and 87.5% and specificities of 92.9% and 98.2%, respectively.

## 7. Conclusions

The radiological diagnosis of peritoneal carcinomatosis of gastric origin is a challenge. It is essential to have a good clinical orientation and to personalize the management of each patient. As none of the existing imaging tools are ideal, imaging has to be adapted depending on patient condition, tumour pathology, and therapeutic situation. A combination of techniques, such as thoraco-abdomino-pelvic CT with abdomino-pelvic MRI for pre-operative assessment, can be a helpful option. Imaging features of peritoneal carcinomatosis from GC can be subtle, so a systematic analysis of the peritoneal cavity is mandatory. This requires a good knowledge of the mechanisms of peritoneal dissemination and a consideration of the anatomical location of the primary tumour, both of which can help in predicting the pattern of peritoneal dissemination. Systematic reading, attention to detail, and experience are all essential for an optimal interpretation. The assessment of treatment response is also complicated due to post-treatment changes. Identification of risk factors for peritoneal recurrence after complete surgical resection is important in order to make decisions regarding the therapeutic approach for the patient. There is no universal consensus on how to carry out radiological follow-up, nor is there consensus on its timing. Regarding new diagnostic techniques, there are promising results in studies with PET-MRI and novel molecules for GC targeting, including FAPI PET/CT and FLT PET/CT, which appear to be interesting for treatment response monitoring and evaluation of diffuse type of GC, respectively. Moreover, radiomics and deep learning are under investigation for the prediction of occult peritoneal metastases in GC. An experienced and dedicated radiologist, as well as a close collaboration between the radiologist, pathologist, oncologist and oncologic surgeon, are essential elements in this multidisciplinary challenge.

## Figures and Tables

**Table 1:** Imaging features associated with an increased risk of incomplete tumour resection, according to Sugarbaker [32].

Bowel obstruction or partial obstruction at more than one site
Mesentery drawn together by tumour (clumped bowel)
Tumour infiltrating between leaves of the small bowel mesentery
Tumour ≥ 5 cm in diameter in jejunal regions
Mesenteric or para-aortic lymphadenopathy
Hydroureter
Psoas muscle invasion
Pelvis sidewall invasion
Seminal vesicle invasion
Hepatoduodenal ligament infiltration and/or bile duct obstruction
Tumour ≥ 5 cm in diameter in gastrohepatic ligament or subpyloric space
Gastric outlet obstruction

## Data Availability

Not appliable.
